# Development of two species of the *Trypanosoma theileri* complex in tabanids

**DOI:** 10.1186/s13071-022-05212-y

**Published:** 2022-03-21

**Authors:** Alexei Yu. Kostygov, Alexander O. Frolov, Marina N. Malysheva, Anna I. Ganyukova, Daria Drachko, Vyacheslav Yurchenko, Vera V. Agasoi

**Affiliations:** 1 Zoological Institute of the Russian Academy of Sciences, St. Petersburg, 190121 Russia; 2 Life Science Research Centre, Faculty of Science, University of Ostrava, 71000 Ostrava, Czech Republic; 3 Martsinovsky Institute of Medical Parasitology, Sechenov University, Moscow, 119435 Russia; 4 Natural-Geographical Faculty, Pskov State University, Pskov, 180000 Russia

**Keywords:** Trypanosomes, Life cycle, Vector, Horseflies, Deerflies

## Abstract

**Background:**

*Trypanosoma theileri* species complex includes parasites of Bovidae (cattle, sheep, goat, etc.) and Cervidae (deer) transmitted mainly by Tabanidae (horse flies and deerflies) and keds (Hippoboscidae). While morphological discrimination of species is challenging, two big clades, TthI and TthII, each containing parasites isolated from bovids and cervids, have been identified phylogenetically. To date, the development in the vector has been studied in detail only for the ked-transmitted sheep parasite *T. melophagium* (TthII), while the fate of trypanosomes in tabanids was described only briefly by light microscopy.

**Methods:**

We collected infected tabanids of various species and identified trypanosomes by molecular phylogenetic analysis. The morphology and development of trypanosomes was studied using the combination of statistical analyses as well as light and electron microscopy.

**Results:**

Two trypanosome species belonging to both TthI and TthII clades of the *T. theileri* complex were identified. The phylogenetic position of these two trypanosomes suggests that they parasitize deer. Both species were indiscernible by morphology in the vector and showed the same development in its intestine. In contrast to the previously described development of *T. melophagium*, both trypanosomes of tabanids only transiently infected midgut and settled mainly in the ileum, while pylorus and rectum were neglected. Meanwhile, the flagellates developing in the tabanid ileum (pyriform epimastigotes and metacyclic trypomastigotes) showed similarities to the corresponding stages in *T. melophagium* by morphology, mode of attachment to the host cuticle and formation of the fibrillar matrix surrounding the mass of developing parasites. In addition, for the first time to our knowledge we documented extraintestinal stages in these trypanosomes, located in the space between the epithelium and circular muscles.

**Conclusions:**

The development of different species of flagellates of the *T. theileri* complex in their insect vectors shows many similarities, which can be explained not only by their common origin, but also the same transmission mode, i.e. contamination of the oral mucosa with the gut content released after squashing the insect either by tongue or teeth. The observed differences (concerning primarily the distribution of developmental stages in the intestine) are associated rather with the identity of vectors than the phylogenetic position of parasites.

**Graphical Abstract:**

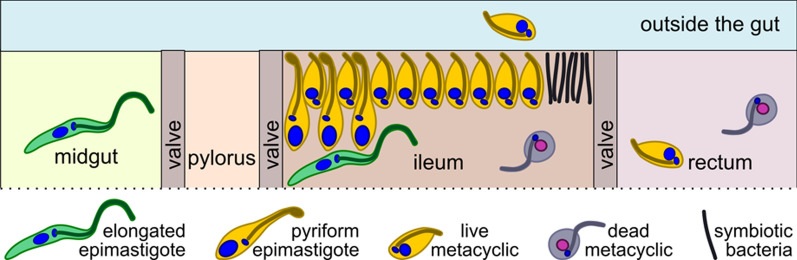

**Supplementary Information:**

The online version contains supplementary material available at 10.1186/s13071-022-05212-y.

## Background

*Trypanosoma theileri* is one of the first trypanosome species described in mammals [1]. It was originally characterized in cattle from Transvaal (now Republic of South Africa) and later documented in various bovines throughout the world, often under different names, which were subsequently synonymized with *T. theileri* (reviewed in [2]). Similar trypanosomes have also been described from other ruminants: *T. melophagium* from sheep, *T. theodori* from goat, *T. ingens* from antelopes and cattle, as well as *T. mazamarum*, *T. cervi*, *T. stefanskii*, and *T. trinaperronei* from various cervids [2–6]. It is not clear how reliable the discrimination of all these species was, given that most of them differed by size characters and trypanosomes are known to manifest pleomorphism (significant variation in size and shape during development) [2]. However, cross-infection experiments demonstrated that deer trypanosomes are not infective to cattle, and, vice versa, the flagellates from cows cannot settle in cervids [4, 7].

The large size of these trypanosomes (40–100 µm) was considered a distinctive feature and justified their separation (together with similar parasites from other hosts, e.g. monkeys and bats) into the subgenus *Megatrypanum* with *T. theileri* as the type species [8]. This trait was indeed important to discriminate these flagellates from other trypanosomes of livestock, such as *T. vivax*, *T. congolense*, and *T. brucei* ssp. causing severe diseases collectively named African animal trypanosomiasis. In contrast, species of the subgenus *Megatrypanum* are typically considered non-pathogenic [2]. Nevertheless, there are multiple reports that *T. theileri* can be an opportunistic pathogen acting in synergy with viruses or piroplasmids, or even be able to cause mild to severe symptoms alone, sometimes leading to death in fetuses and neonate calves [9–18].

Phylogenetic analyses performed using several molecular markers on a wide range of *Megatrypanum* isolates from various ruminants and blood-sucking dipterans demonstrated that although this group is monophyletic, it consists of several lineages united into two big clades, TthI and TthII, each containing parasites isolated from bovids and cervids [6, 19–24]. Thus, the whole group is considered *T. theileri* species complex [25], within which only *T. melophagium* and recently described *T. trinaperronei* can be identified with certainty. For other species of the complex including *T. theileri*, it is currently impossible until a scrutinous taxonomic revision is performed.

Most trypanosomes of the complex are transmitted by Tabanidae (horseflies and deerflies), while keds (Hippoboscidae) serve as vectors for *T. melophagium*, *T. trinaperronei*, and *T. theodori* [2, 6]. *Trypanosoma theileri*-like trypanosomes were also documented in various mosquitoes, a sandfly, and a tsetse fly [26–28]. In addition, *T. theileri* has been repeatedly reported in ticks [29–32]. However, the only study assessing the phylogenetic position of a trypanosome from a cattle-associated tick demonstrated that it is phylogenetically distant from *Megatrypanum* and is closely related to the poorly studied *T. pestanai* clade [33]. Experiments with transmission of *T. melophagium*, as well as trypanosomes of cattle and deer, have demonstrated that infection of mammalian hosts is achieved only when vector gut content is applied to the oral mucosa (which does not need to be damaged), while routes known in other trypanosomes (through a bite or via abraded skin) are ineffective [7, 34, 35].

Studies of the development of *Megatrypanum* from ruminants in vectors started at the beginning of the twentieth century, even before it became clear that the flagellates in the guts of keds and tabanids are trypanosomes [36–38]. Later, there was an attempt to investigate the development of *T. theileri* in tabanids with light microscopy using parasite-free insects fed on an infected cow [39]. Thus, the sequential colonization of different gut sections and transformation of cell types have been observed, but details of the host-parasite relationships and the ultrastructure of trypanosomes have not been described. A similar study has been performed for *T. theodori* vectored by the goat ked *Lipoptena capreoli* [2]. More attention has been paid to *T. melophagium*, whose development in the sheep ked has been studied in detail using light as well as transmission and scanning electron microscopy. This illuminated the distribution of parasites across the gut, continuity of the developmental stages, modes of cell attachment, etc. [40–42]. It is furthermore surprising that the main vectors of *T. theileri-*like trypanosomes remained virtually neglected.

In this work, we described the development of two species belonging to the TthI and TthII clades of the *T. theileri* complex in tabanids using light and electron microscopy and demonstrated that it is similar in both cases, although distinct from that of *T. melophagium.* In addition, for the first time, we documented presence of extraintestinal stages of these parasites in insect vectors.

## Materials and methods

### Hosts collection, dissection, and isolation of trypanosome-containing gut sections

**Fig. 1:**
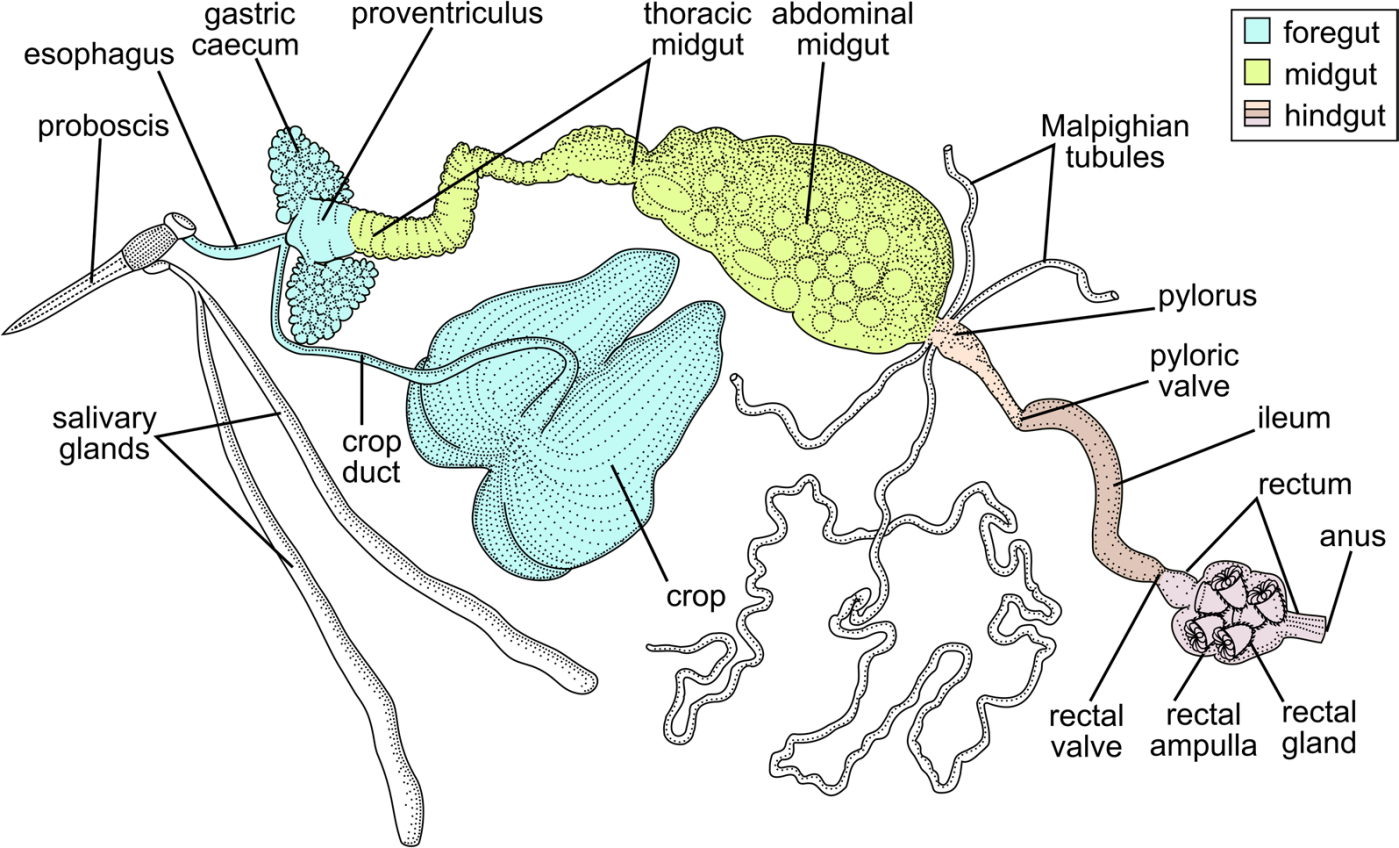
Generalized structure of the digestive system in Tabanidae

Tabanids attacking the authors of this work were manually collected into individual vials with water-containing tubes in 2018, 2020, and 2021 in four locations in the Northwestern Federal District of Russia (Table 1). Within 24 h after their capture, the insects were killed with chloroform and dissected in normal saline. The entire digestive tube (Fig. 1) was isolated, carefully placed on a slide, covered with a cover glass, and inspected under light microscope. After detection of infection, the corresponding parts of the intestine were used for smear preparation, DNA isolation, or electron microscopy.

**Table 1:** Isolates of trypanosomes studied in this work

Isolate	Species	Host (vector)	Locality	Year
194Tab	Tthα	*Hybomitra solstitialis*^b^	Karelia, Lakhdenpokhya town (61°31ʹN, 30°12ʹE)	2018
KrSL1^a^	Tthβ	*Hybomitra tarandina*		
KrSL4^a^	Tthα	*Hybomitra muehlfeldi*		
KrSL7^a^	Tthα	*Chrysops divaricatus*		
513SL	Tthβ	*Hybomitra muehlfeldi*	Leningrad Oblast, Bol'shoye Rakovoye Lake (60°37ʹN, 29°22ʹE)	2020
519SL	Tthβ			
D1011	Tthβ	*Hybomitra bimaculata*	Leningrad Oblast, Toksovskoye village (60°09ʹN, 30°35ʹE)	2021
D1012	Tthα	*Hybomitra muehlfeldi*		
D1013	Tthα	*Chrysops viduatus*		
D1016	Tthα	*Hybomitra solstitialis*^b^		
D1017	Tthα	*Hybomitra bimaculata*		
F1187	Tthβ	*Hybomitra nitidifrons confiformis*	Novgorod Oblast, Oksochi village (58°39ʹN, 32°47ʹE)	2021
F1206	Tthα			

^a^Isolated in a previous study [43]

^b^*H. solstitialis* (Meigen, 1820) = *H. ciureai* (Séguy, 1937)

### DNA isolation, PCR and sequencing

Infected intestine fragments, preserved in the solution containing 1% SDS and 50 mM EDTA after dissection, served to identify trypanosomes. Total DNA was isolated from these samples with the DNeasy Blood & Tissue Kit (Qiagen, Hilden, Germany) following the manufacturer’s protocol. This DNA was used to specifically amplify either the nearly full-length trypanosomatid 18S rRNA gene with the primers S762 and S763 [44] or its 836-bp fragment (encompassing variable regions V8, V3, V4 and V9) with the primer pair 1127F (5ʹ-aggcattcttcaaggataccttcc-3ʹ) and 1958R (5ʹ-tgatgagctgcgcctacgaga-3ʹ) [45]. A ~ 900-bp fragment of the glycosomal glyceraldehyde-3-phosphate dehydrogenase (gGAPDH) gene was amplified using the primers G3 and G4a [46]. PCR fragments were sequenced using either amplification primers or, in the case of the full-length 18S rRNA gene, a set of internal primers as described before [47]. Samples showing mixed signal were excluded from subsequent analyses. The sequences were deposited in GenBank under accession numbers OL855997–OL856006 (18S rRNA gene) and OL860973–OL860974 (gGAPDH gene).

### Phylogenetic analyses

The nucleotide sequences obtained in this study along with related ones retrieved from GenBank were aligned by MAFFT v. 7.475 [48] using the E-INS-i and L-INS-i algorithms for the 18S rRNA and gGAPDH genes, respectively. The 18S rRNA alignment was deduplicated to preserve the longest available sequence for each haplotype, while keeping information about all sequences belonging to these haplotypes. A maximum likelihood tree was inferred in IQ-TREE v. 2.1.3 [49] under the K2P + I substitution model selected by the built-in ModelFinder module [50]. Edge support was estimated using the ultrafast bootstrap method with 1000 replicates. The phylogeny inference using the gGAPDH gene was performed by the maximum likelihood method in IQ-TREE and the Bayesian approach in MrBayes v. 3.2.7 [51] under the partitioned model (F81 + F + I, JC + I and TIM2 + F + G4 selected by ModelFinder for the first, second and third codon positions, respectively). Branch lengths were unlinked among the three character sets. Edge support in IQ-TREE was assessed with 1000 standard bootstrap replicates. The analysis in MrBayes was run for 10 million generations with every 100th sampled and other parameters set by default.

### Microscopy and morphometry

The ethanol-fixed smears prepared during tabanid dissection were stained with Giemsa, examined under light microscope, photographed and measured as described earlier [52]. Observed cells were classified into three main morphotypes, for which six standard morphometric features were evaluated: cell length (not including the free flagellum) and width, nucleus length, distances between the anterior end and the nucleus or kinetoplast, and free flagellum length. The results of these measurements are presented in Additional file 1: Table S1. The morphometric characters (except for the length of free flagellum that could not be reliably measured in all cells) were used for comparison of morphotypes between trypanosome species and individual isolates within a particular species by principal component analysis (PCA) in PAST 4.08 software using default settings [53]. The results were visualized as two-dimensional plots with the raw data projected onto axes representing eigenvectors extracted from calculated correlation matrices.

Sample preparation procedures for transmission electron microscopy (TEM) have been described earlier [54]. For scanning electron microscopy (SEM), glutaraldehyde-fixed fragments of tabanid guts were air dried on coverslips, which were subsequently attached to specimen stubs, coated with gold, and examined with Tescan MIRA3 LMU electron microscope at 5 kV (Tescan, Brno, Czech Republic). These analyses were performed for the isolates 194Tab, 513SL, 519SL (TEM) and D1011-D1013, D1016 and D1017 (SEM).

To study the distribution of trypanosomes in the hindgut in detail, the 194Tab sample processed for (TEM) was also used to prepare serial semithin (700 nm) sections. The latter were placed on glass slides in a drop of water, then attached by drying on a warm stage at 60 °C and stained there with Richardson stain [55] for 30–60 s.

## Results

### Molecular phylogenetic analyses

**Fig. 2:**
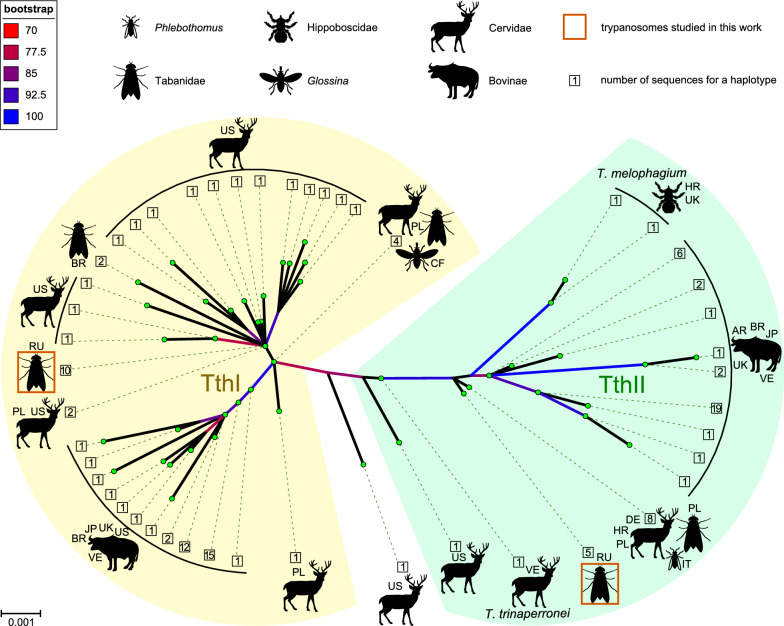
Unrooted maximum likelihood tree based on 18S rRNA gene sequences of the *Trypanosoma theileri* complex. Pictograms show the source of isolates from which sequences have been obtained. Two-letter codes indicate countries of origin following the scheme of national internet domains: *AR* Argentina, *BR* Brazil, *CF* Central African Republic, *DE* Germany, *HR* Croatia, *IT* Italy, *JP* Japan, *PL* Poland, *RU* Russia, *UK* United Kingdom, *US* United States, *VE* Venezuela. Scale corresponds to the number of substitutions per site. Ultrafast bootstrap supports are shown by branch coloring only for values > 70. The borders of the TthI and TthII clades are marked according to [6]. GenBank accession numbers of the sequences used are available in Additional file 2: Fig. S1, representing the same tree in a traditional rectangular format with labels

The 18S rRNA gene sequences of trypanosomes from the infected gut specimens represented only two variants (haplotypes), which we have observed in a previous study [43]. These two variants were unambiguously associated with the *Trypanosoma theileri* complex, and we provisionally named them Tthα and Tthβ. The difference between the haplotypes consisted of eight substitutions and one 2-nt indel, considering the whole length of the 18S rRNA gene corresponds to < 0.5% difference. The phylogenetic analysis demonstrated that Tthα and Tthβ belong to two different clades of the *T. theileri* complex—TthI and TthII, respectively (Fig. 2, Additional file 2: Fig. S1). Within the TthI lineage, the Tthα species showed affinity to the cluster composed of sequences from cervids and tabanids, although the support of this cluster is low. Moreover, almost identical (differing in just a single 1-nt indel) sequences were obtained from a white-tailed deer in the USA and a tabanid in Poland. These facts suggest that Tthα is a deer trypanosome. As for Tthβ, its position on the 18S rRNA gene-based tree does not allow assessing the affinity of this trypanosome, although the most similar sequences were obtained from European cervids, tabanids and a sandfly.

**Fig. 3:**
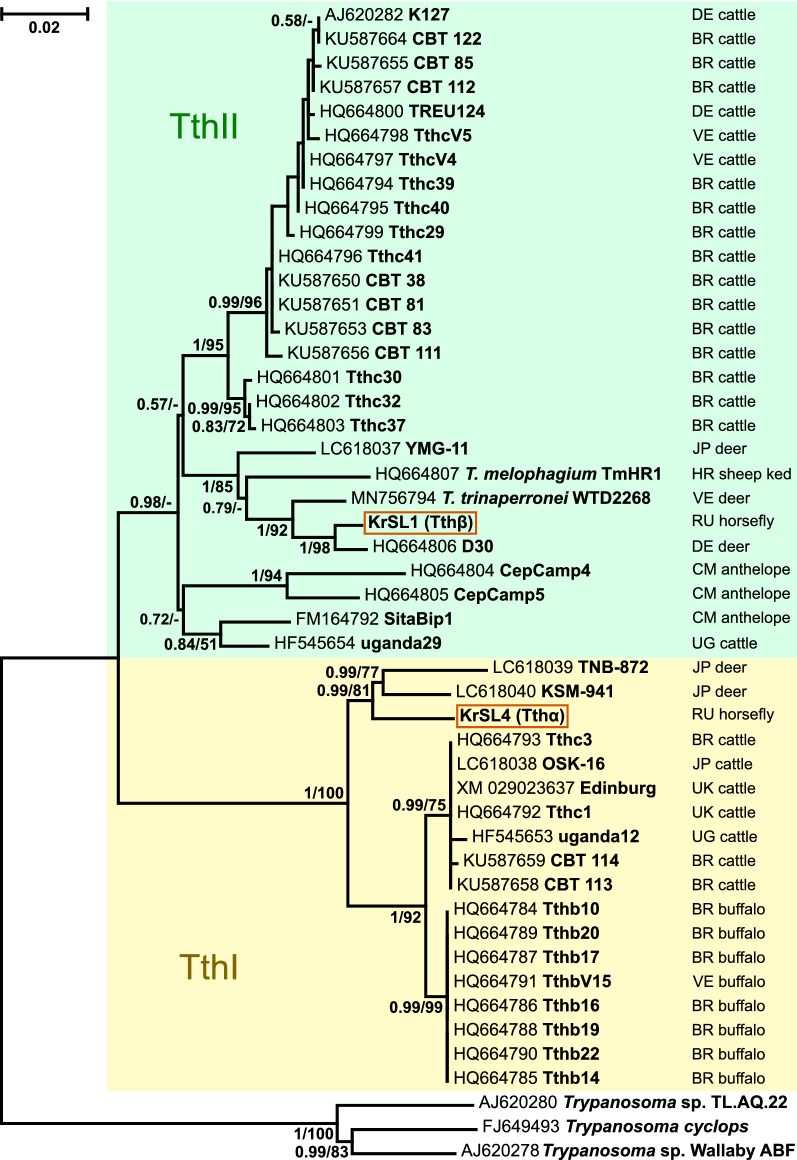
Maximum likelihood phylogenetic tree of *Trypanosoma* (*Megatrypanum*) spp. based on gGAPDH gene sequences. Bayesian posterior probabilities and bootstraps are shown at branches for values > 0.5 and 50, respectively. Two-letter codes indicate countries of origin following the scheme of national internet domains (*BR* Brazil, *CM* Cameroon, *DE* Germany, *JP* Japan, *RU* Russia, *UG* Uganda, *UK* United Kingdom, *US* United States, *VE* Venezuela). Scale corresponds to the number of substitutions per site. The borders of the TthI and TthII clades are marked according to [6]. Three sequences of trypanosomes not belonging to the *T. theileri* complex are used as outgroups (not highlighted)

Although the 18S rRNA gene has the advantage of a large database of sequences, it typically does not provide sufficient resolution when dealing with related species. Therefore, we applied another marker, the gGAPDH gene, which better resolves relationships between *Trypanosoma* spp. [46, 56, 57]. Indeed, the inferred tree helps to position the two trypanosomes under study with high statistical support (Fig. 3). Tthα again clusters with cervid trypanosomes, more specifically with those from Japanese sika deer (these isolates are not present on the 18S rRNA gene-based tree). The close relationship of Tthβ with the abovementioned group of European isolates [represented here by the isolate D30 (from a fallow deer), whose sequences are available for both genes] becomes unambiguous. Of note, the recently described *T. trinaperronei* from white-tailed deer is the next closest relative.

### Light microscopy

**Fig. 4:**
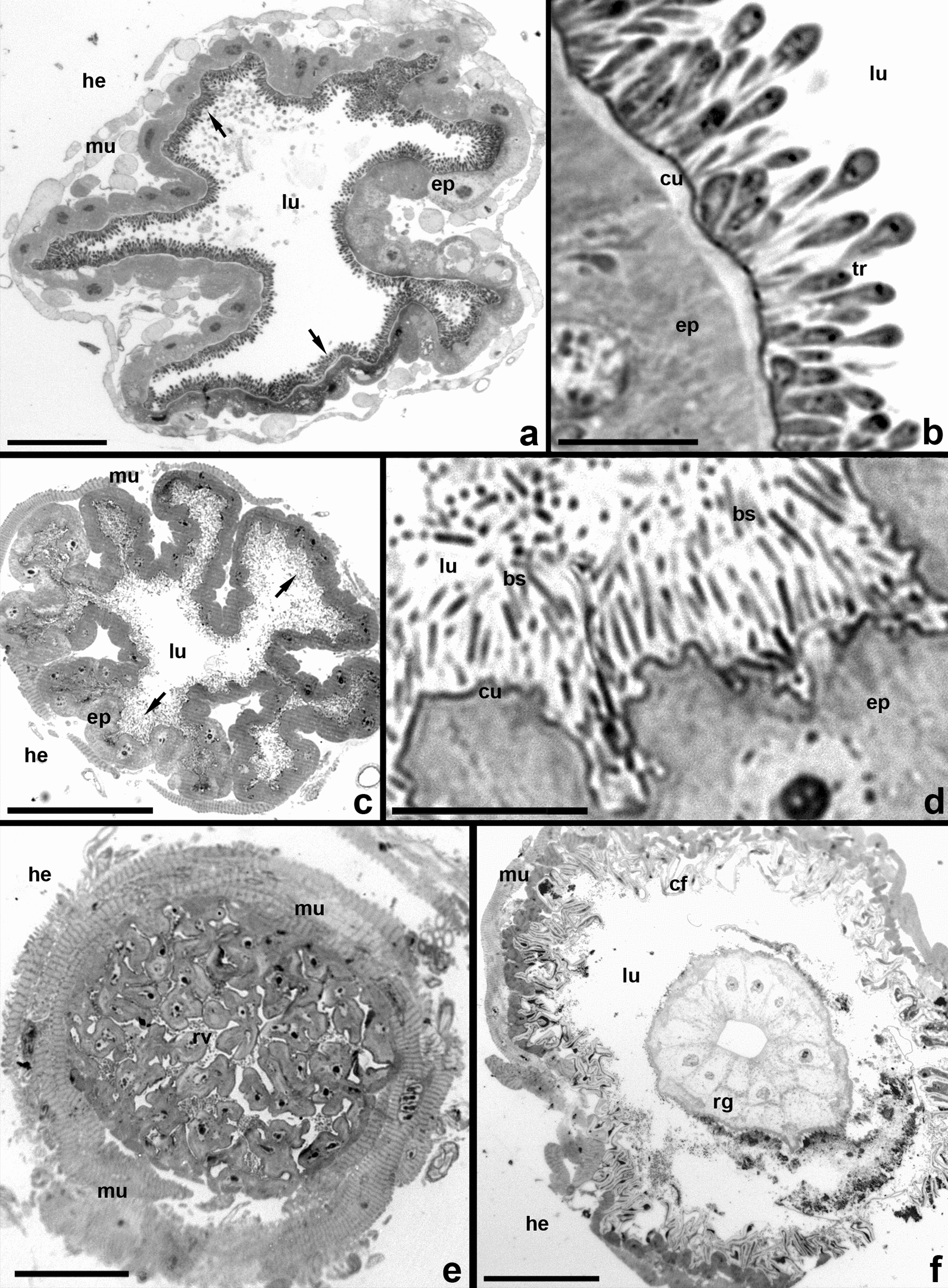
Semithin cross sections of the hindgut (isolate 194Tab). Richardson stain. **a**, **b** Trypanosomes on the ileum cuticle. **c**, **d** Posterior portion of ileum, containing only symbiotic bacteria. **e** Rectal valve. **f** Rectal ampulla with one of the six rectal glands. *bs* bacterial symbionts, *cf* cuticle folds, *cu* cuticle, *ep* intestinal epithelium, *he* hemocoel, *lu* intestinal lumen, *mu* muscles of the intestine, *rg* rectal gland, *tr* trypanosome cells. Arrows in **a** and **c** show trypanosomes and bacteria, respectively. Scale: **a** 50 µm; **b**, **d** 10 µm; **c**, **e** 40 µm; **f** 100 µm

In all infected tabanids, trypanosomes were always present in the ileum, while in the midgut and rectum no or a few flagellates could be observed. Localization of trypanosomes in the hindgut was studied in detail for isolate 194Tab using a series of semithin sections (Fig. 4).

At most of the length of the ileum, the majority of trypanosomes are attached to the intestinal wall, completely covering its inner surface in 2–3 layers (Fig. 4a, b). However, they do not settle near the border with the rectal valve, where the ileum forms 8–10 large diverticula containing symbiotic bacteria. The latter are localized either on the cuticular lining of the epithelium or in the diverticular lumina (Fig. 4c, d). The rectal valve and the rectal ampulla, which contains six large glands, are also free of trypanosomes (Fig. 4e, f).

**Fig. 5:**
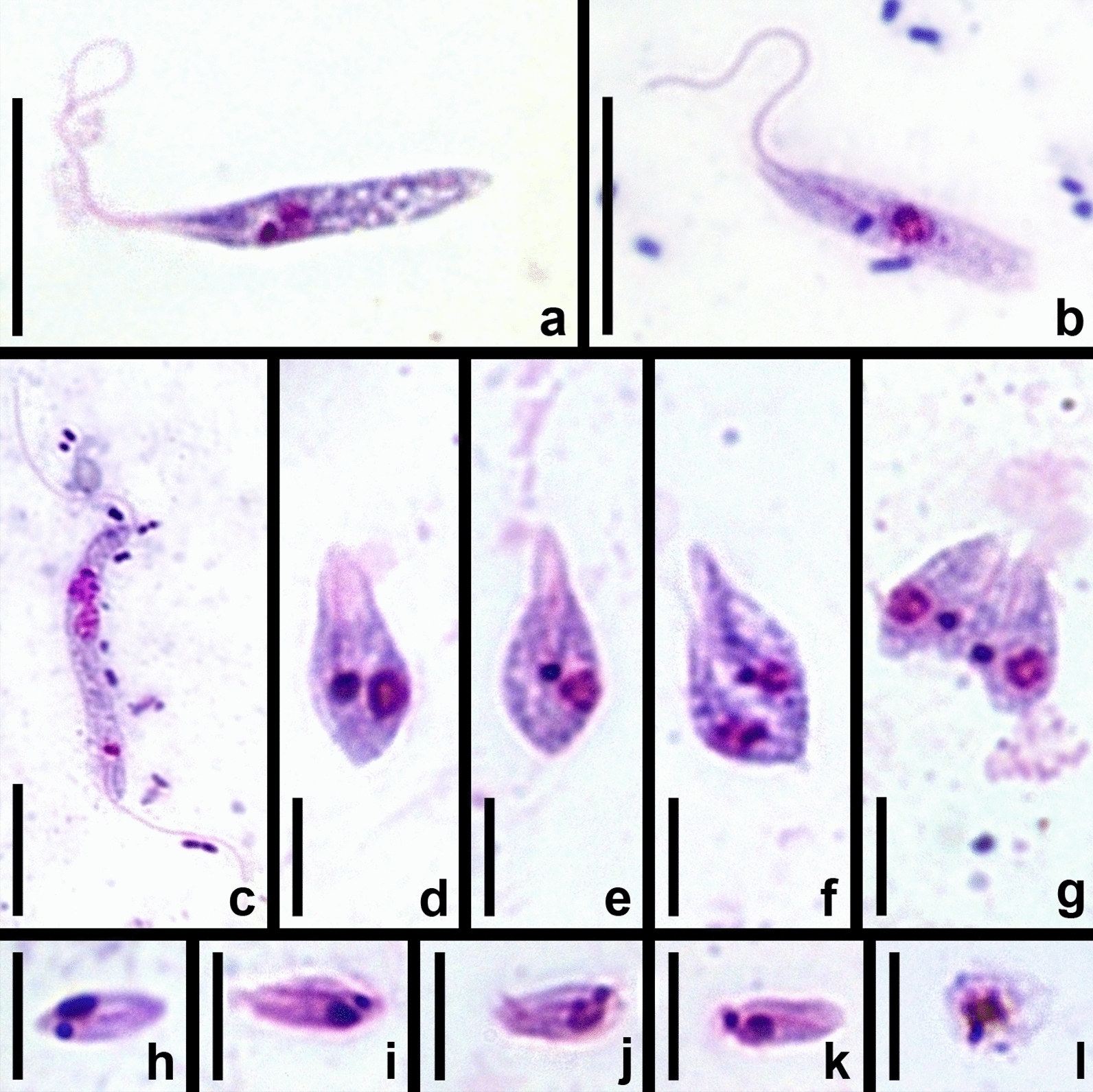
Morphotypes of trypanosomes observed in the hindgut of tabanids (Giemsa-stained smears). **a**, **b** Non-dividing elongated epimastigotes. **c** Dividing elongated epimastigotes. **d**, **e** Non-dividing pyriform epimastigotes. **f**, **g** Dividing pyriform epimastigotes. **h**–**k** Metacyclics. **l** Dead cell from the rectum. **a**, **d**, **i** 513SL (Tthβ); **b**, **c** F1206 (Tthα); **e**, **g**—KrSL7 (Tthα); **f**, **h**—KrSL1 (Tthβ); **j**, **k**, **l**—194Tab (Tthα). Scale: **a**–**c** 10 µm; **d**–**l** 5 µm

The micropopulation of trypanosomes in the ileum is heteromorphic and consists of three main morphotypes (proportions increase from first to last (Table 2): free elongated epimastigotes (Fig. 5a–c), attached pyriform epimastigotes (Fig. 5d–g) and attached metacyclic trypomastigotes (Fig. 5h–k). Only epimastigotes of both types are able to proliferate (Fig. 5c, f, g). In elongated epimastigotes, the kinetoplast is localized near the anterior margin of the nucleus, which is situated in the anterior third of the cell (Fig. 5a–c). In pyriform epimastigotes, the nucleus and kinetoplast are shifted to the center of the cell but preserve their mutual arrangement (Fig. 5d, e). The products of their division can preserve the same features (Fig. 5f) or become more similar to metacyclics, with the nucleus displaced backward and the kinetoplast migrating to its posterior margin (Fig. 5g). In metacyclic trypomastigotes, representing the predominant morphotype, the nucleus has a subcaudal position, while the kinetoplast is placed either near the posterior margin of the latter or behind it (Fig. 5h–k).

**Table 2:** Proportions of cell morphotypes in the ileum

Isolates	Tthα				Tthβ	
	KrSL7	F1206	D1017	194Tab	KrSL1	513SL
Elongated epimastigotes	1.46%	2.80%	3.15%	0.56%	0.14%	0.65%
Pyriform epimastigotes	13.99%	16.78%	5.14%	8.09%	4.12%	7.67%
Metacyclics	84.55%	80.42%	91.71%	91.35%	95.74%	91.68%
*N*	686	143	603	686	2934	1082

**Fig. 6:**
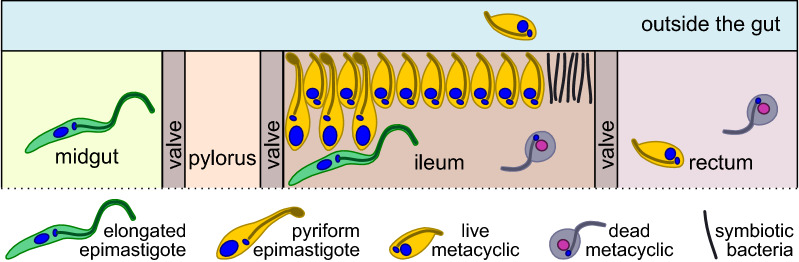
Scheme of the distribution of observed cell types in the intestine of tabanids

Rare trypanosomes observed in the midgut are exclusively represented by elongated epimastigotes. In the rectum, in addition to occasional metacyclics, dead rounded cells can be observed. On Giemsa-stained smears, these cells show signs of degradation: broken contour, “effloresced” and often vacuolized cytoplasm, etc. (Fig. 5l). Of note, such cells can also be observed in the ileum. The distribution of trypanosomes in different gut sections is summarized in Fig. 6.

**Fig. 7:**
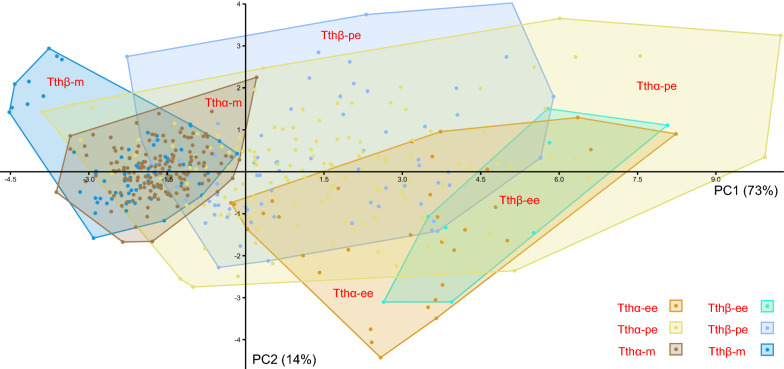
Scatterplot of the principal component analysis showing a two-dimensional space of morphometric data in three cell morphotypes of two trypanosome species: Tthα (isolates F1206, D1017, KrSL7, and 194Tab) and Tthβ (isolates 513SL and KrSL1). PC1 and PC2: principal components 1 and 2, respectively; numbers in parentheses show the percentage of variance explained by a particular component. *ee* elongated epimastigotes, *pe* pyriform epimastigotes, *m* metacyclics

Despite some variation in size and shape within each of the three cell types, we did not detect any visible differences between the two species of trypanosomes studied here. To check it statistically, we estimated standard morphometric parameters of the three cell types in six isolates belonging to both species (Additional file 1: Table S1) and subjected them to principal component analysis. Over 87% of variance could be explained by the two principal components with the strongest contribution of cell length followed by distances between the anterior end and nucleus or kinetoplast (Additional file 3: Table S2). Confirming the previous assessment by eye, the species Tthα and Tthβ could not be reliably discriminated by any of the morphotypes (Fig. 7). However, such a difference could be observed between different isolates of a single species (Additional file 4: Fig. S2). Furthermore, in agreement with the intermediate status of the pyriform epimastigotes, they showed the largest variation and significant overlap with elongated epimastigotes and metacyclics (Fig. 7).

### Electron microscopy

**Fig. 8:**
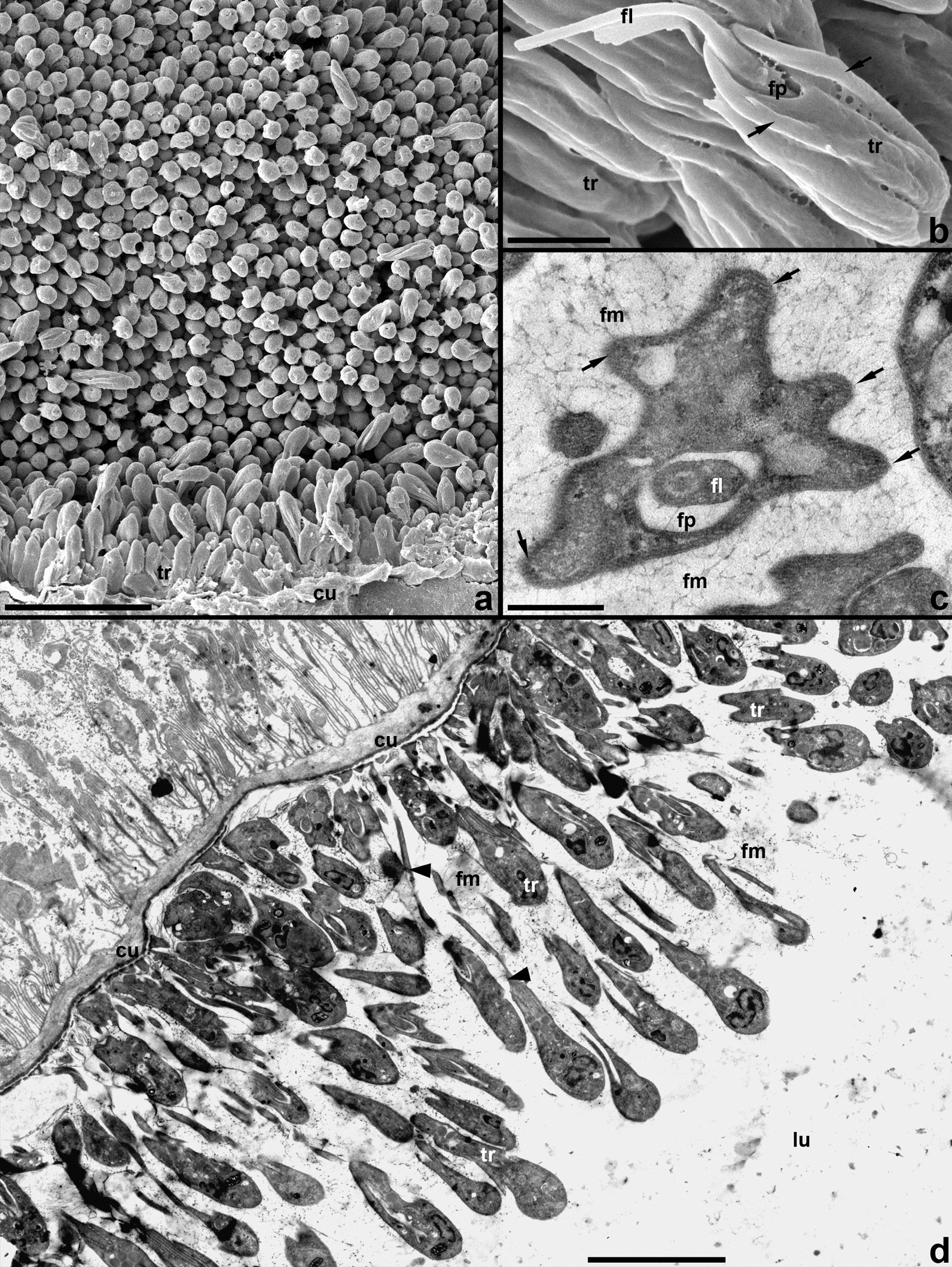
Trypanosomes occupying the cuticular lining of ileum. **a**, **b** SEM; **c**, **d** TEM. **a**, **d** General view. **b** Lateral view of a single cell with broken flagellum. **c** Cross section of a trypanosome cell. Arrows: longitudinal ridges; arrowheads: long flagella of cells from distal rows; *cu* cuticle, *fl* flagellum, *fm* fibrillar matrix surrounding trypanosomes, *fp* flagellar pocket, *lu* intestinal lumen, *tr* trypanosomes. Scale: **a** 10 µm, **b** 1 µm, **c** 0.4 µm, **d** 4 µm

**Fig. 9:**
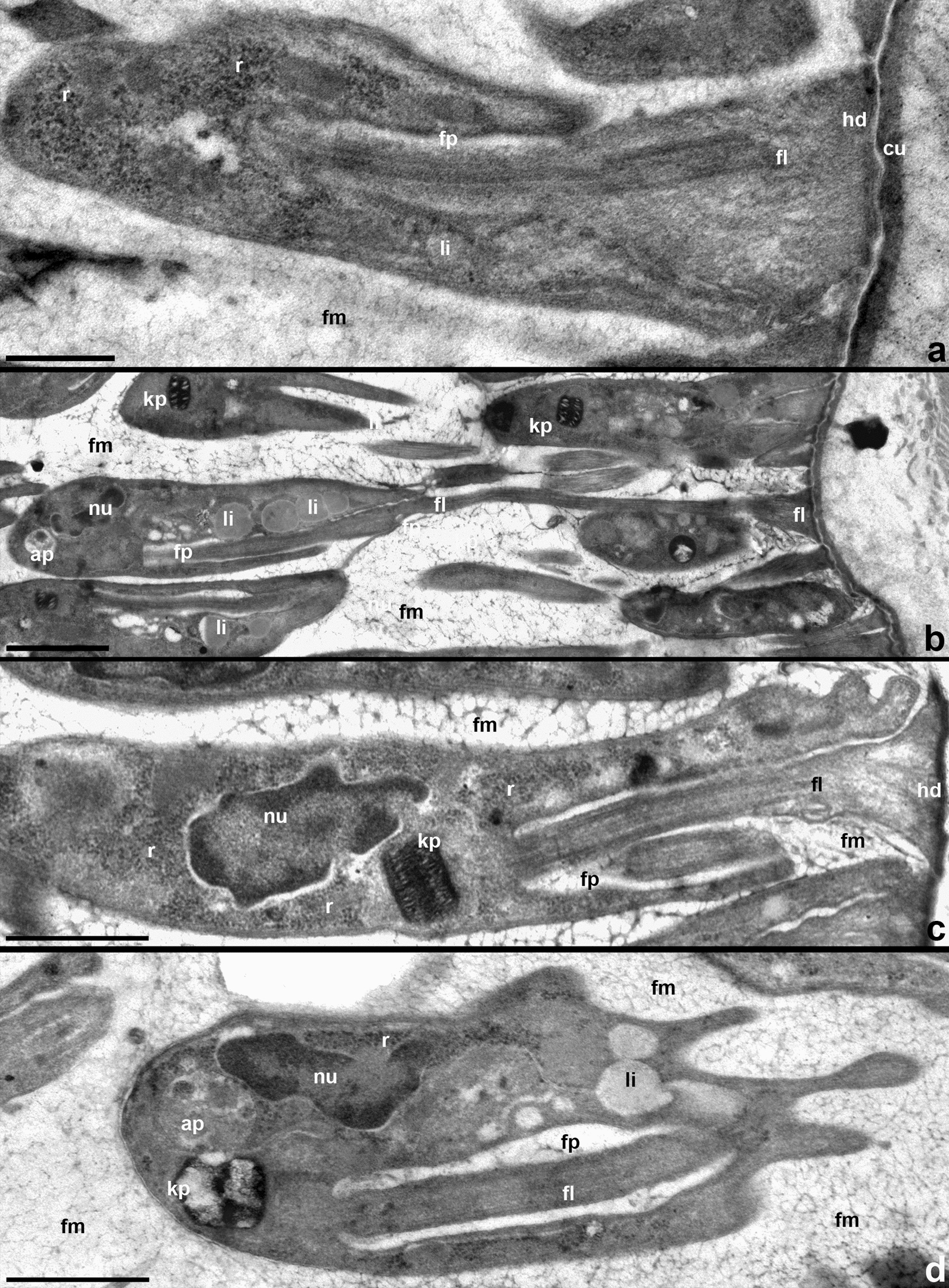
Fine structure of trypanosomes in the ileum. **a**–**c** Pyriform epimastigotes. **d** Metacyclic. Cells are attached using widened tips of short (**a**, **b**, **c**) or long (**b**) flagella with a zonal hemidesmosome and surrounded by fibrillar matrix. *ap* autophagosome, *cu* cuticle, *fl* flagellum, *fm* fibrillar matrix, *fp* flagellar pocket, *hd* hemidesmosome, *kp* kinetoplast, *li* lipid droplet, *nu* nucleus, *r* ribosomes. Scale: **a** 0.6 µm; **b**, **c** 2 µm; **d** 0.8 µm

The details described in this section were observed in both Tthα and Tthβ; therefore, no reference to particular isolates or species is provided. The epithelium of the infected ileum is covered with 2–3 layers of flagellates tightly adjoining each other (Fig. 8a, d). The surface of trypanosome cells forms longitudinal ridges (Fig. 8b, c). The parasites are attached to the cuticular lining of the ileum using modified flagellar tips, which form a sucker-like thickening (Figs. 8d and 9a–c). The cells of the first (proximal) row bear very short flagella, widening before the exit from the flagellar pocket (Fig. 9a, c). A zonal hemidesmosome is formed under the plasmalemma of flagellar tips in the area of contact with the cuticle (Fig. 9a, c). Trypanosomes in distal rows use flagella of a length that allows them to attach directly to the cuticle (Figs. 8d and 9b). Therefore, despite the high density of parasites near the surface of the intestinal wall, each of them is attached individually and no specific contacts between neighboring cells are detected. However, the trypanosomes are submerged in a matrix made of fibrils with a diameter of 3–5 nm, which form a loose network associated with the plasmalemma of flagellates and fill gaps between them and the cuticular lining of the ileum (Figs. 8c, d and 9a–d). This fibrillar matrix has an integrative function, and therefore those cells that have lost connection to the intestinal wall remain in the common mass of parasites.

Trypanosomes developing in the ileum rarely display acidocalcisomes, while glycosomes could not be detected at all. Instead, their cytoplasm typically contains vacuoles with electron-light content (consistent with lipids) in the anterior half of the cell and autophagosomes in the posterior one (Fig. 9b, d). Attached epimastigotes and mature metacyclics show ultrastructural differences. The cytoplasm of the former is rich in ribosomes, which fill the space between the main cell compartments more or less evenly (Fig. 9a, c). In metacyclics, ribosomes are sparse and concentrate near the nucleus and at the cell periphery (Fig. 9d). In epimastigotes, the laterally opening flagellar pocket is short (Fig. 9a, c), while in metacyclics it extends throughout most of the cell length (Fig. 9d). These two cell types also show differences in the organization of the kinetoplast. In epimastigotes, it has a nearly rectangular profile, which measures on average 0.56 × 0.28 µm and demonstrates a well-discernible network of circular DNA (Fig. 9c). In metacyclics, the kinetoplast has a barrel-shaped profile measuring on average 0.54 × 0.43 µm. Its DNA forms condensed islands in the periphery and center, which are interconnected by looser “bridges” (Fig. 9d).

**Fig. 10:**
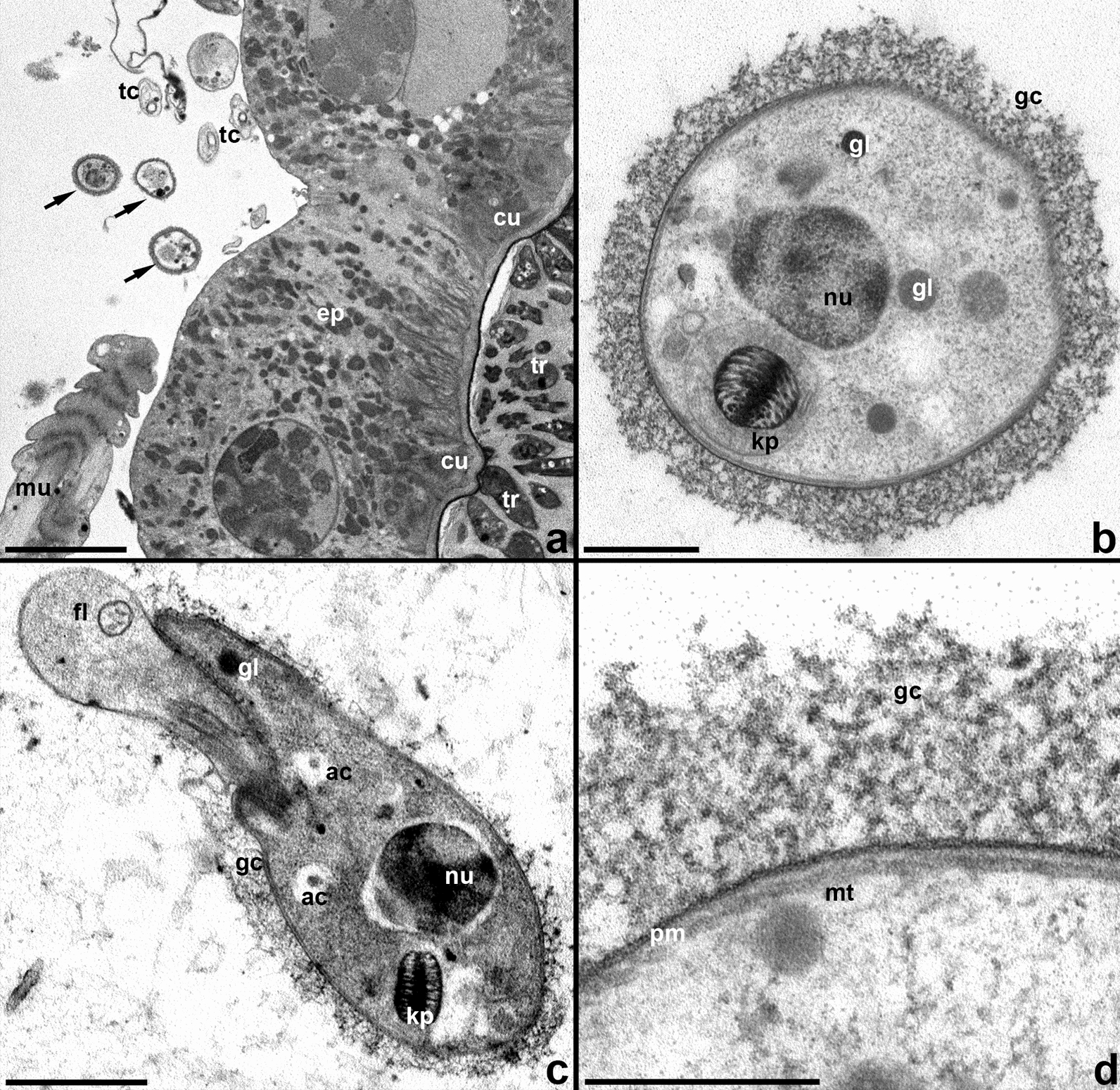
Extraintestinal trypanosome cells. **a** Trypanosomes from both sides of the intestinal wall. **b**, **c** Single cells, cross and longitudinal sections, respectively. **d** Cell coverings structure. *ac* acidocalcisome, *cu* cuticle, *ep* intestinal epithelium, *fl* flagellum, *gc* glycocalyx, *gl* glycosome, *kp* kinetoplast, *mt* microtubules, *mu* muscles, *nu* nucleus, *pm* plasmalemma, *tc* tracheoles, *tr* trypanosomes in the gut lumen. Arrows point to extraintestinal trypanosomes. Scale: **a** 6 µm, **b** 0.4 µm; **c** 1 µm; **d** 0.2 µm

Trypanosomes can also be found on the outside of the ileum wall, between the intestinal epithelium, which is not underlain by a basal membrane, and circular muscles. In the space, enclosed between these two layers and containing tracheoles and longitudinal muscles, single cells or small groups of parasites are localized (Fig. 10a). These trypanosomes do not contact any host tissues or each other. The surface of their plasmatic membrane harbors a fibrillar glycocalyx developed to varying degrees, with thickness reaching 200 nm (Fig. 10b, d). Individual fibrils of this glycocalyx have the same diameter as those in the matrix surrounding the intestinal forms, i.e. 3–5 nm. In contrast to the cells observed in the lumen of the ileum, the overwhelming majority of extraintestinal stages display an even cell surface (Fig. 10a, b). The arrangement of the nucleus and kinetoplast in these cells indicates that they are metacyclic trypomastigotes (Fig. 10b, c). However, they differ from intestinal metacyclics by the presence of acidocalcisomes and glycosomes, short club-shaped flagellum and the organization of kinetoplast reminiscent of that in epimastigotes (Fig. 10b, c).

## Discussion

In this work, we studied the development of the parasites belonging to two different species of the *Trypanosoma theileri* complex, which, in the absence of information on the morphology of their bloodstream forms, were provisionally designated as Tthα and Tthβ. Although nesting within two different clades of the complex (TthI and TthII), these two species are highly similar. Both were found in tabanid vectors and, as judged by our phylogenetic inferences, apparently represent the parasites of cervids. We were unable to discriminate these species by morphology, morphometry, ultrastructure or development in the vector. It remains unclear whether they have any differences in the vertebrate host or represent classical twin species.

Although Tthα and Tthβ do not show differences from each other, their development in the intestine of the vector is clearly distinct from that described for *T. melophagium* in the sheep ked *Melophagius ovinus.* The latter was shown to colonize the midgut, where elongated epimastigotes attach to the brush border of the epithelium by intertwining their flagella with microvilli and actively multiply. Then, they spread to the pylorus, ileum and rectum, attaching to the cuticle of these hindgut sections as proliferative pyriform epimastigotes and non-proliferative metacyclics [40–42]. The trypanosomes studied here do not form a stable micropopulation in the midgut (Fig. 6). Its colonization is transitory, and the reason certainly lies in the absence of attached forms within this intestinal section. This is in line with a previous study, showing that epimastigotes in the midgut of tabanids could be observed only on days 1–4 post-infection [39]. In contrast to *T. melophagium*, Tthα and Tthβ settle only in the ileum, ignoring the pylorus and rectum (Fig. 6). The absence of these flagellates from the latter section is especially surprising, since it is one of the preferred locations for trypanosomatids, many of which live on the surface of the rectal glands/pads or in close proximity to them [58]. This distinction may be explained by differences in the physiology of the digestive system in keds and tabanids. Regrettably, to the best of our knowledge, such studies have not been performed so far. In line with this assumption are the data on *T. theodori*, although its affiliation to the *T. theileri* complex has not been verified using molecular methods so far. The development of this trypanosome in the goat ked resembles that of *T. melophagium*, except for the presence of trypomastigotes (along with epimastigotes) in the midgut [2]. The mechanism of transmission in *T. theodori* is the same as in *T. melophagium* and very similar to that in tabanid-transmitted flagellates: contamination of oral mucosa with intestinal content released after squashing the insect either by tongue (in cows and deer [7, 34]) or teeth (in sheep and goats [2]).

One more important difference between *T. melophagium* and the trypanosomes studied here concerns the contacts between the flagella of attached forms in the hindgut, which allow them to form rosettes. The connection between cells is preserved even when such a rosette breaks away from the intestinal wall [40, 41]. These contacts were never observed in the trypanosomes from tabanids studied here. However, another mechanism that helps the attached cells to stay together, namely the formation of the fibrillar matrix surrounding the entire mass of parasite cells attached in the hindgut, is present in all three species of trypanosomes under discussion. Previously, it has been argued that this matrix is of trypanosome origin, since it appears only in the hindgut, the cuticular lining of which prevents secretion of complex molecules [59]. Here we demonstrated that the fibrils forming the matrix are similar to those that constitute the glycocalyx layer on the surface of the cells from extraintestinal location, confirming that they are produced by the parasites.

Our detection of trypanosomes outside the ileum lumen is especially intriguing. In contrast to other trypanosomatids that traverse the intestinal wall, because it is an indispensable part of their development within the host [60–62], the exit process could not be captured here, and we observed only its result. Furthermore, the number of parasite cells located from outside of the intestinal epithelium was always modest, further indicating that this may occur inadvertently. We hypothesize that this phenomenon is occasional and occurs after an accidental rupture of the intestinal wall, which then quickly self-heals [63]. The cells do not appear to die there, since we observed a transformation of their ultrastructure, suggesting some further development. However, they were never detected in the process of division and, therefore, may represent another kind of persistent stage, which is distinct from the intestinal metacyclics. The observed morphological changes may be related to the fact that these cells no longer need to be attached. It is not possible to judge whether the same phenomenon exists in the case of *T. melophagium*, since extraintestinal locations were not specifically assessed for that species and, due to the sparsity of the cells outside the gut, it is not possible to detect them with light microscopy except for semithin sections (Additional file 5: Fig. S3).

## Conclusions

We demonstrated that the development of flagellates of the *T. theileri* complex in vectors depends on the identity of the latter rather than on the phylogenetic position of the former. However, in general, all these trypanosomes develop similarly, which can be explained by virtually the same mode of transmission.

## Supplementary Information

**Additional file 1: Table S1.** Summarized cell measurements.

**Additional file 2: Figure S1.** Detailed 18S rRNA gene-based tree of *T. theileri*-like trypanosomes listing accession numbers of sequences used. New sequences are marked with asterisks.

**Additional file 3: Table S2.** Loadings and proportion of variance for the two principal components.

**Additional file 4: Figure S2.** Scatterplot of the principal component analysis showing a two-dimensional space of morphometric data in three cell morphotypes individually for each isolate. PC1 and PC2: principal components 1 and 2, respectively, numbers in parentheses show the percentage of variance explained by a particular component. ee = elongated epimastigotes, pe = pyriform epimastigotes, m = metacyclics.

**Additional file 5: Figure S3.** Extraintestinal trypanosome cells on semithin sections. A:isolate 513SL, B: isolate 519SL.

## Data Availability

The sequences obtained in this study were submitted to GenBank and are available under accession numbers OL855997–OL856006 (18S rRNA gene) and OL860973–OL860974 (gGAPDH gene).

## Abbreviations

EDTA: Ethylenediaminetetraacetic acid
gGAPDH: Glycosomal glyceraldehyde 3-phosphate dehydrogenase
SEM: Scanning electron microscopy
SDS: Sodium dodecyl sulfate
TEM: Transmission electron microscopy
