# Supplementary figures and images for: Development of two species of the Trypanosoma theileri complex in tabanids
Source: Parasit Vectors. 2022 Mar 21;15:95. doi: 10.1186/s13071-022-05212-y (PMC8935851; doi:10.1186/s13071-022-05212-y)

# TthI

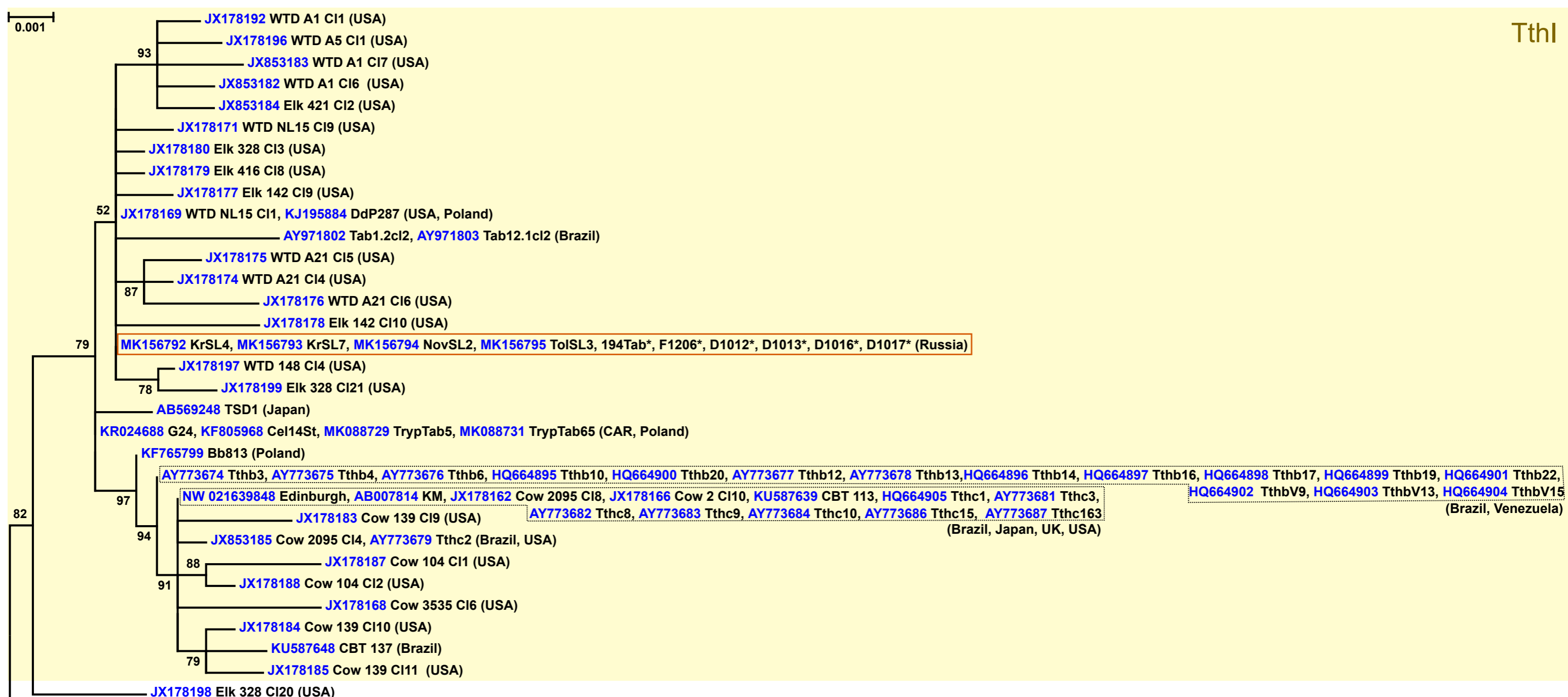

## TthII

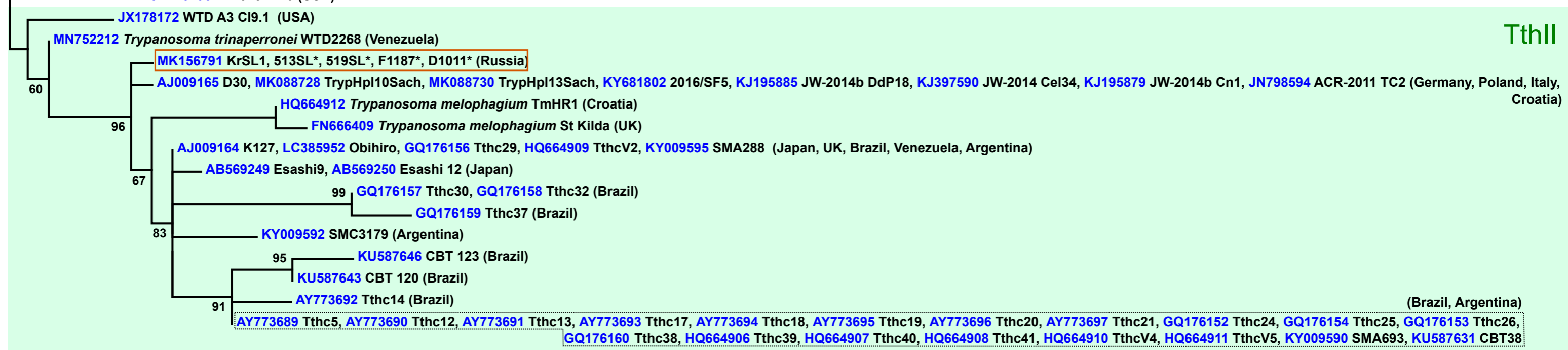

Supplement: Supplementary file 2 — Additional file 2: Figure S1. Detailed 18S rRNA gene-based tree of T. theileri-like trypanosomes listing accession numbers of sequences used. New sequences are marked with asterisks. [file 13071_2022_5212_MOESM2_ESM.pdf]

**Ttha**

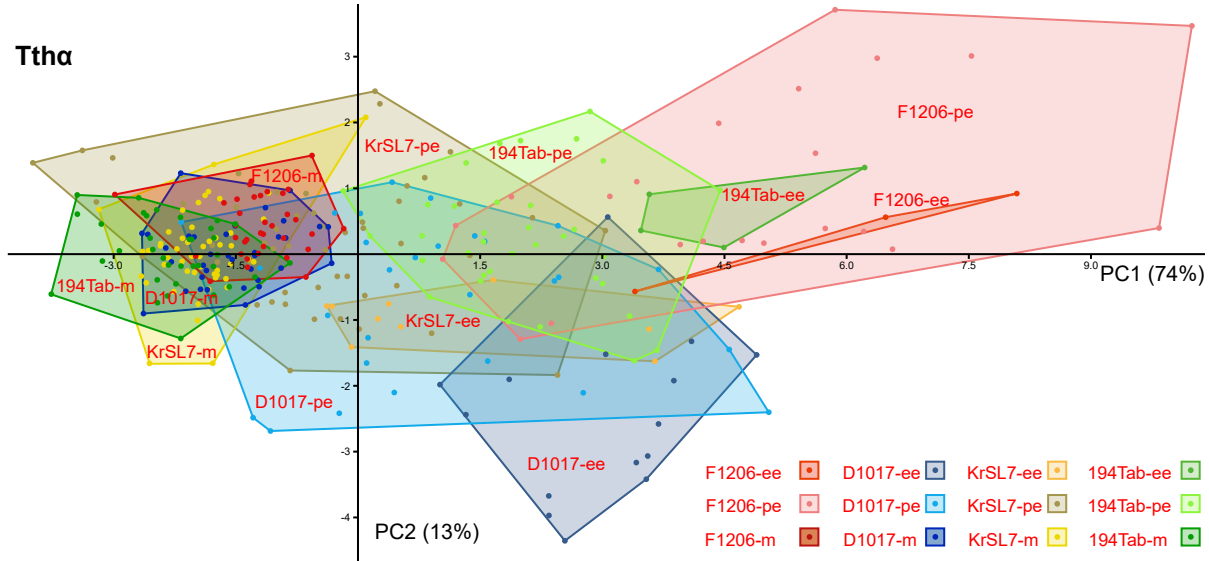

**Tth $\beta$**

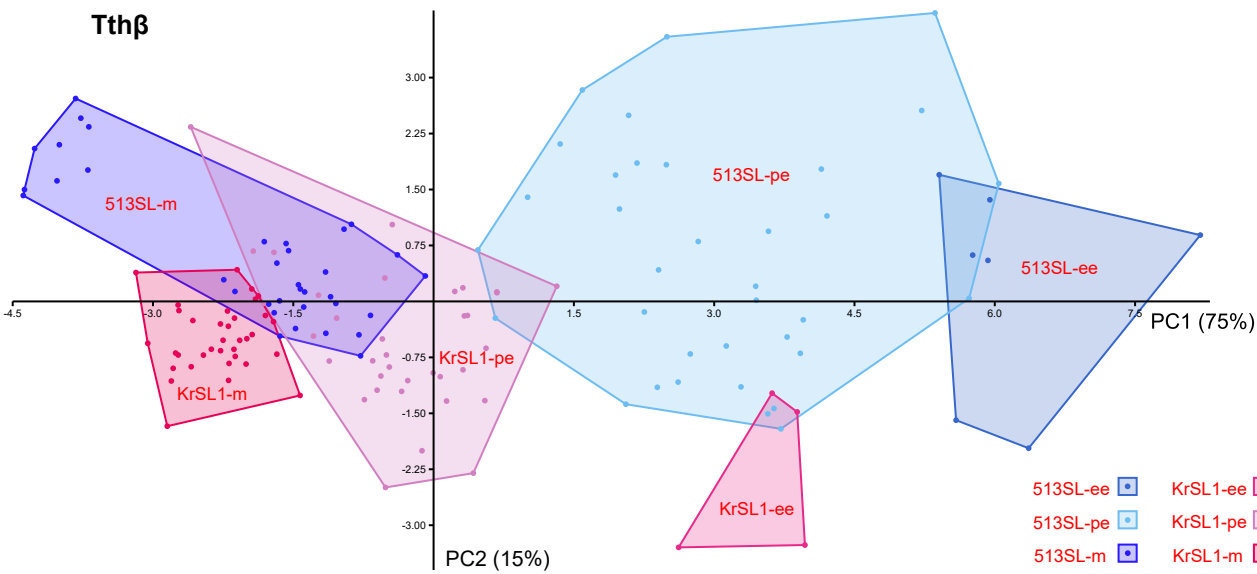

Supplement: Supplementary file 4 — Additional file 4: Figure S2. Scatterplot of the principal component analysis showing a two-dimensional space of morphometric data in three cell morphotypes individually for each isolate. PC1 and PC2: principal components 1 and 2, respectively, numbers in parentheses show the percentage of variance explained by a particular component. ee = elongated epimastigotes, pe = pyriform epimastigotes, m = metacyclics. [file 13071_2022_5212_MOESM4_ESM.pdf]

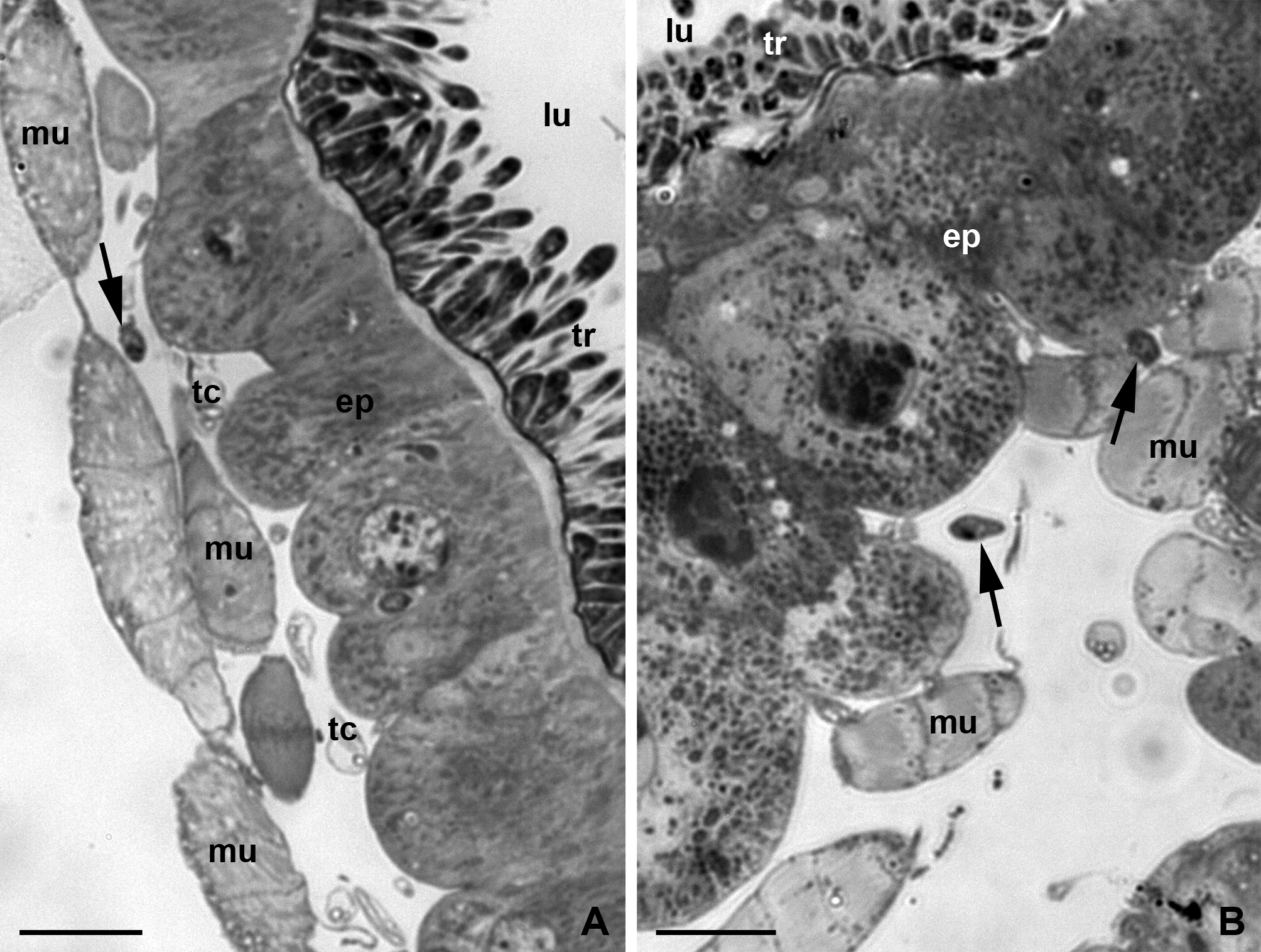

Supplement: Supplementary file 5 — Additional file 5: Figure S3. Extraintestinal trypanosome cells on semithin sections. A:isolate 513SL, B: isolate 519SL. [file 13071_2022_5212_MOESM5_ESM.tif]
